# Evaluation of Histomorphological Parameters to Predict Occult Nodal Metastasis in Early-Stage Oral Squamous Cell Carcinoma

**DOI:** 10.5146/tjpath.2021.01566

**Published:** 2022-09-15

**Authors:** Rahul Verma, Ashok Singh, Nilotpal Chowdhury, Prashant Pranesh Joshi, Prashant Durgapal, Shalinee Rao, Sanjeev Kishore

**Affiliations:** Department of Pathology, All India Institute of Medical Sciences, Rishikesh, India

**Keywords:** Oral squamous cell carcinoma, Nodal metastasis, Histomorphological parameters, Early-stage tumors

## Abstract

***Objective:*** The oral squamous cell carcinoma (OSCC) treatment protocol depends upon lymph node metastasis. Elective neck dissection for early-stage OSCC (pT1/T2) elective neck dissection reduces the morbidity rate. It also reduces the overall survival and thus it becomes important to detect lymph node metastasis in early-stage OSCC.

***Material and Method:*** Various histomorphological parameters have been studied to predict nodal metastasis in early-stage OSCC. We aim to evaluate these parameters in the context of nodal metastasis. 78 cases of early-stage OSCC were included in the study with histopathologic parameters like tumor size, grade, tumor depth of invasion (DOI), lymphovascular invasion (LVI), perineural invasion (PNI), worst pattern of invasion (WPOI), and lymph node level.

***Results:*** Out of the 78 patients, 32 patients had lymph node metastasis. T stage, DOI, LVI, and WPOI showed statistically significant deviance from the null model (P-values of 0.007, 0.01, 0.04 and 0.02 respectively). The Odds Ratio (OR) of T stage, DOI, LVI and WPOI were 4.45 (95% C.I =1.47-14.1), 4.4 (95% C.I =1.32-15.88), 8.12 (95% C.I =1.002-198.20), and 3.39 (95% C.I =1.24-9.74) respectively. On multivariate analysis (Firth logistic regression) using DOI, LVI, and WPOI as independent variables, only T-stage and WPOI retained statistical significance.

***Conclusion:*** The prognostic information supplied by evaluating DOI, LVI, and WPOI warrants the inclusion of these parameters in the standard reporting format for all cases of OSCC.

## INTRODUCTION

Oral cancer is the eighth most common malignancy in the world and third most common in India with an incidence rate of 12.6 per 100000 population (1). Oral squamous cell carcinoma (OSCC) accounts for almost 90% of the oral malignancies (2). The multifactorial etiology ranges from tobacco chewing to genetics. It is also twice as common in males as compared to females with a high mortality rate and overall 5-year survival rate of 50%, which further decreases to 20-36% with nodal metastasis (3,4). According to the National Comprehensive Cancer Network (NCCN) Head and Neck Cancer guidelines, the treatment modality for OSCC varies from resection of the primary with or without the neck dissection. Neck dissection is recommended in advanced stages like T3 and T4. However, there is disagreement over the approach for early-stage OSCC i.e. T1-2, N0. Although neck dissection in early-stage OSCC reduces the morbidity, it also exposes the patient to unnecessary neoadjuvant therapy and overtreatment. On the other hand, primary resection without neck dissection may lead to increased overall survival compared to the elective neck dissection group (5). Thus it becomes the utmost necessity to detect lymph node metastasis, especially in early-stage OSCC. Depth of invasion (DOI) is considered to be the best predictor of occult metastatic disease. It is recommended that clinical judgment should be utilized for cases having DOI of 2-4 mm and elective neck dissection should be done for DOI >4 mm (6). Other histological parameters like the worst pattern of invasion (WPOI), tumor differentiation, T-stage, extra-nodal extension (ENE), lymphovascular invasion (LVI), and perineural invasion (PNI) are also being studied as predictors of local and distant occult metastasis, thus collaborating in the overall decision-making for elective nodal dissection and further helping in reducing the incidence of treatment failure (7,8). These histological parameters can be used to create a predictive model to ascertain the risk of metastasis in early-stage OSCC and further aiding in treatment decision-making. Various risk prediction models have been suggested by the authors to predict the nodal metastasis in the early as well as late-stage OSCC (9–11). Most of the scores and systems are being validated in the western countries only with an exception to the Brandwein Gensler risk model which was validated in the Indian scenario by Chaturvedi et al (9). A new scoring system, the Aditi-Nuzhat Lymph-node Prediction Score (ANLPS) System, was developed by Arora et al. exclusively on the Indian population for early-stage OSCC (11). Our study aims to analyze various histomorphological parameters of early-stage OSCC as predictors of occult nodal metastasis.

## MATERIAL and METHODS

**Figure 1:**
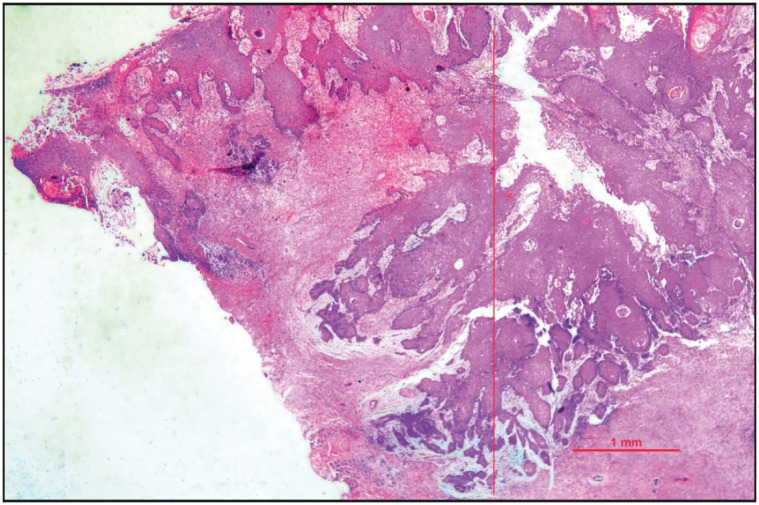
Depth of invasion (DOI) measurement in oral squamous cell carcinoma (OSCC). Histomorphological picture showing DOI of 3mm in an OSCC pathological stage T2 (H&E stain, 2x magnification)

**Figure 2:**
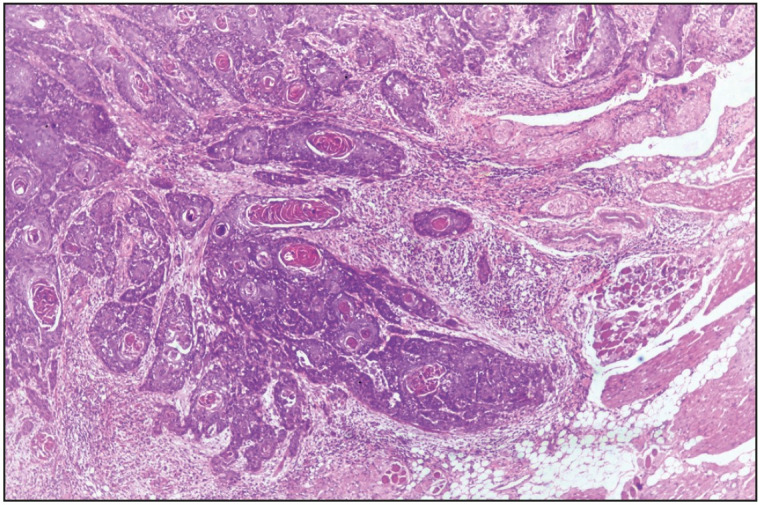
Histomorphological picture showing WPOI-5 an OSCC pathological stage T2 (H&E stain, 4x magnification)

**Figure 3:**
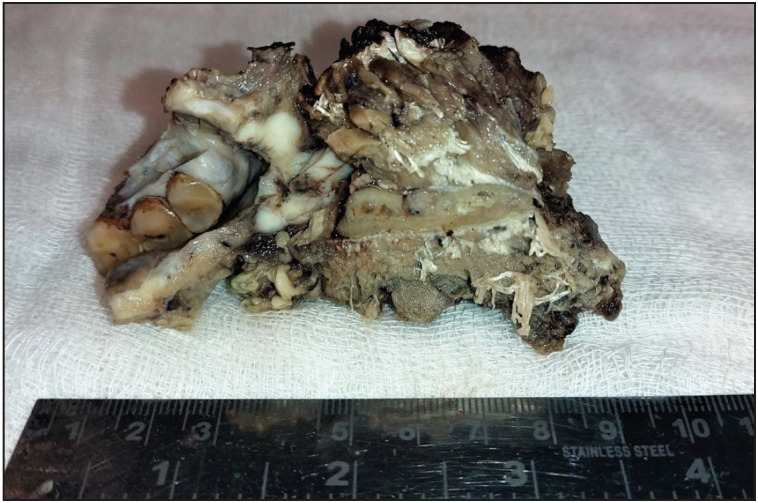
Gross mandibulectomy specimen showing OSCC pT2 stage.

This retrospective analytical study was conducted in the Department of Pathology, in a tertiary care hospital in North India, by reviewing the archival data of all the OSCC patients who went for primary tumor resection with or without neck node dissection during the period of one year from January 2019 to December 2020. Patients with biopsy- proven OSCC and pathological stage pT1/T2 were included in the study. Patients with prior neoadjuvant therapy, recurrence, multiple tumors, verrucous carcinoma, and incomplete data were excluded from the study. After recording the demographic and clinical details, the cases were reviewed by two pathologists independently for the following parameters: tumor size, histopathological tumor grade (well, moderate and poorly differentiated), pathological tumor stage (TNM), DOI, regional lymph node metastasis along with the cervical level, LVI, PNI, and WPOI. On histology, the tumor was graded as described by Que et al (12). Tumors showing easily recognizable squamous epithelium, abundant keratinization, intercellular bridges, minimal pleomorphism, and basally located mitotic figures were graded as well-differentiated (Grade 1). Those tumors where squamous lineage was difficult to determine, having none or minimal keratinization and marked nuclear atypia were graded as poorly differentiated (Grade 3), and tumor cells having features in between well and poorly differentiated grades with focal keratinization and pearl formation were graded as moderately differentiated (Grade 2.) DOI was measured in millimeters ( mm) using an eyepiece graticule micrometer from the basement membrane of adjacent normal to the deepest point of invasion (Figure 1). WPOI was graded as 1-4 and 5. WPOI 1-4 included tumors having pushing borders (WPOI 1), finger-like tumor borders (WPOI 2), large islands of >15 tumor cells/ island close to <1 mm to the main tumor (WPOI 3), small islands of <15 tumor cells/ island close to <1 mm to the main tumor and dispersed tumor satellites >1 mm away from the main tumor (WPOI 5, Figure 2) (13). WPOI 1-4 tumors are non-aggressive and thus were kept in one subgroup. TNM Staging was done according to the American Joint Committee on Cancer (AJCC) classification. Figure 3 shows a mandibulectomy specimen having OSCC reported as pathological T-stage 2. The level of lymph nodes involved was also noted for creating the prediction model of nodal metastasis.

### Statistical Analysis

Statistical analyses were performed using R software (v 3.6.0) (14), with help from the packages “exact2x2” (15), “pROC” (16), and “logistf” (17). Descriptive analysis for clinical factors was done with continuous variables described as Mean ±standard deviation and categorical variables described as proportion. For univariate analysis of the histopathological parameters with nodal status, the significance of the association between dichotomous categorical variables (LVI, WPOI, T stage: stage 1 vs. stage 2, DOI dichotomized into two groups: less than 4 cm and greater than or equal to 4 cm) and node status was estimated by the usual Fisher’s exact test and matching confidence interval (15) of the conditional odds ratio by the “exact2x2” R package. For 2x6 tables (e.g. relationship between tumor site and lymph node status), the generalized Fisher’s exact test was used. The univariate analysis of DOI as a continuous variable was also done by univariate Firth logistic regression. The variables that were found statistically significant at an alpha of 0.05 were further studied in a multivariate analysis using Firth penalized logistic regression and profile penalized log-likelihood confidence intervals (18). The assumption of linearity in the logit for the Firth regression was tested by the Box-Tidwell test. We subsequently performed a ROC curve analysis for the continuous variables significantly associated with nodal metastasis on univariate analysis. Finally, Mann-Whitney-Wilcoxon tests and Fisher exact tests were used to examine the relationship between the variables found significant in univariate analysis. All tests were 2-sided with significance considered at p<0.05.

## RESULTS

The baseline demographics and clinicopathological data of a total of 189 cases were recorded (Table 1). The mean age of the patients comprising 170 (89.9%) males and 19 (10.1%) females was 48±12.9 years. Buccal mucosa (n=95) (50.2%) was the most common primary site in the oral mucosa followed by the tongue (n=60) (31.7%). Most of the cases were moderately differentiated histological grade n=112(59.2%) and T4a pathological stage n=74(39.6%). A total of 90(47.6%) cases were found to be nodal positive with maximum cases (n=25) (27.7%) falling in the N1 pathological stage. A total of 78 cases were found to be of the pT1/T2 stage. These cases were included in the study for further analysis.

**Table 1:** Baseline demographics and clinicopathological data of all cases.

**Variable**	**Value**
Patients (N)	189
Age (Mean±sd)	48±12.9 years
Gender Male Female	170 (89.9%) 19 (10.1%)
Tumor site Alveolar Process Buccal Mucosa Gingivo-buccal sulcus Lip Maxilla Palate Paranasal sinus Retromolar Tongue	17 (8.9%) 94 (50.2%) 6 (3.1%) 3 (1.5%) 2 (1%) 4 (2.1%) 1 (0.5%) 1 (0.5%) 61 (31.7%)
Histological Grade Well differentiated Moderately differentiated Poorly differentiated	75 (39.6%) 112 (59.2%) 2 (0.01%)
pT* stage T1 T2 T3 T4a T4b	26 (13.7%) 53 (28%) 33 (11.6%) 74 (39.6%) 1 (0.5%)
pN* (Total positive) N1 N2 N2a N2b N2c N3 N3b	93 (49.2%) 25 (26.8%) 3 (3.3%) 4 (4.3%) 28 (30.1%) 8 (8.6%) 2 (2.1%) 22 (23.6%)
Tumor size, cm [Mean (SD)]	3.7 (2.6)
DOI, mm (mean (SD)	12.7 (10.3)
LVI (Total positive)	23 (12.1%)
PNI (Total positive)	32 (16.9%)
WPOI 1-4 WPOI 5	94 (49.7%) 95 (50.2%)

*Pathological TNM staging. **DOI**: Depth of invasion, LVI: Lymphovascular invasion, **PNI**: Perineural invasion, **WPOI**: Worst pattern of invasion.

### Clinical Parameters of T1/T2 Tumors

Table 2 summarizes the results of the clinical and demographic parameters of early-stage OSCC (T1/T2) to evaluate them as predictors of nodal metastasis. None of the parameters was statistically significant to predict the nodal metastasis.

**Table 2:** Demographic and clinical parameters in early-stage OSCC (T1/T2).

**Parameter**	**LN positive(n=32)**	**LN negative(n=46)**	**p value**
Age( Mean±sd)	47.1±12.1 years	46.5±13.7 years	0.85*
Gender Male Female	27 05	44 2	0.12**
Tumor site Alveolar Process Buccal Mucosa Gingivo-buccal sulcus Tongue Lip Maxilla Palate	2 12 2 16 0 0 0	0 22 0 20 1 1 2	0.12***

* t-test **Fisher exact test ***Generalized Fisher exact test.

### Histomorphological Parameters of Early-Stage OSCC (T1/T2)

Table 3 summarizes the association of various histomor-phological parameters with the lymph node status. Out of all the parameters, four parameters composed of the T-stage, DOI, LVI, and WPOI were statistically significant. Other parameters did not show a statistically significant correlation with nodal metastasis in early-stage OSCC (T1/T2). On multivariate analysis, only WPOI and T-stage retained their significance (Table 4), but these results are limited by a small sample size. The Box-Tidwell test did not reveal a statistically significant violation of the assumption of linearity of the logit when using the Firth penalized logistic regression.

**Table 3:** Histomorphological parameters in early-stage OSCC (T1/T2). The statistical significance has been tested by the Fisher exact test, with corresponding Confidence intervals, unless indicated otherwise.

**Parameter**	**Lymph Node positive(n=32)**	**Lymph Node negative(n=46)**	**OR**	**95%CI**	**p-value**
Histological Grade Grade 1 Grade 2 Grade 3	15 17 0	20 26 0	0.87	0.33 -2.33	0.82
pT stage T1 T2	05 27	21 25	4.45	1.47 - 14.1	**0.007**
DOI, mm ≤ 3.99 ≥ 4.00	04 28	18 28	4.4	1.32-15.88	**0.01**
DOI (As continuous variable)			1.14	1.01 - 1.31	0.04*
LVI Present Absent	05 27	01 45	**8.12**	**1.002 - 198.20**	**0.04**
PNI Present Absent	04 28	03 43	2.03	0.41-11.03	0.43
Worst pattern of invasion WPOI 1-4 WPOI 5	12 20	30 16	**3.39**	**1.24-9.74**	**0.02**

*Firth Logistic regression. **DOI**: Depth of invasion, **LVI**: Lymphovascular invasion, **PNI**: Perineural invasion, **WPOI**: Worst pattern of invasion

**Table 4:** Result of multivariate Firth logistic regression done using the variables found to be statistically significant on univariate analysis, with the 95% CI of the odds ratio.

	**Coefficient**	**Odds Ratio (OR)**	**Lower 95% C.I. of OR**	**Upper 95% C.I. of OR**	**p-value**
Intercept	-3.21	0.04	0.004	0.27	0.0006
DOI ( mm)	0.02	1.024	0.88	1.19	0.75
LVI	1.54	4.66	0.67	58.88	0.12
T stage (2 vs 1)	1.29	3.64	1.09	13.54	0.035
WPOI ( 5 vs 4)	1.009	2.74	1.026	7.62	0.044
	Likelihood ratio test=16.18 on 4 df, p=0.002				

**DOI**: Depth of invasion, **LVI**: Lymphovascular invasion, **WPOI**: Worst pattern of invasion

On a ROC curve analysis for DOI, a cut off of 6 mm was found to give the highest sum of sensitivity and specificity, and thus the Youden J. The results of the sensitivity and specificity at the region of highest performance along with the area under the curve are given in Table 5.

**Table 5:** The Specificity and Sensitivity at various thresholds for the ROC curve analysis of the Depth of Invasion (DOI), along with the Area under curve (AUC) with 95% Confidence intervals by the deLong Method.

Threshold	≥3 mm	≥4 mm	≥5 mm	≥6 mm*	≥7 mm
Specificity	0.28	0.39	0.5	0.61	0.65
Sensitivity	0.94	0.87	0.72	0.69	0.59
AUC	0.67 (95% CI: 0.55 - 0.79)				

* Best Threshold by Youden’s J

We also performed tests for the association between the significant factors found in univariate analysis. Among these factors, DOI was significantly associated with the T-stage (P-value <0.001 by the Mann-Whitney-Wilcoxon test) and showed a trend towards significance with LVI (p-value of 0.09 by the Mann-Whitney-Wilcoxon test test). None of the other factors showed a significant association with each other.

## DISCUSSION

The oral cancer burden worldwide is approximately 300000/year out of which India has the highest share (20%). OSCC is the commonest malignancy in the oral cavity with a male to female ratio of 3:1 (19). According to NCCN guidelines, the treatment protocol for OSCC depends upon the TNM staging. The mainstay of the treatment is primary resection with or without neck node dissection. For advanced-stage OSCC, i.e. T3-T4, treatment includes primary resection with neck node dissection followed by neoadjuvant therapy if necessary. However, for early-stage OSCC, i.e. T1-T2, there is an option for either performing elective neck dissection or observing with follow up so that loco-regional recurrence is detected early and salvage surgery can be performed (20). Although OSCC with no clinical nodal involvement rarely (<10%) presents with nodal metastasis, it is imperative to detect occult metastasis in early-stage OSCC. Elective neck dissection provides comprehensive clearance of nodes, increases overall survival rate, and prevents loco-regional recurrences, in addition to increasing the aesthetic and functional morbidity (21). Several authors have compared elective node dissection and follow up observation in early-stage OSCC and found the nodal recurrence rate to be higher in the observation group compared to the elective node dissection group. These patients were further subjected to salvage surgery, which involves aggressive approach and increases mortality (22–24). Determining which approach to follow thus requires multiple large-scale studies and comprehensive reviews.

The various histological parameters are being studied as prognostic factors associated with the survival rate in OSCC (25). We studied the histological parameters like pathological TNM stage, tumor grade, DOI, WPOI, PNI, and LVI.

DOI has been considered as an important prognostic as well as regional nodal involvement parameter in many studies (11,26–28). The National Comprehensive Cancer Network (NCCN) Head and Neck Cancer guidelines suggest elective neck dissection in tumors with DOI greater than 4 mm. DOI is different from the tumor thickness as the former one is measured from the basement membrane between tumor and adjacent normal surface to the maximum depth of the tumor whereas tumor thickness also takes into account the mucosal surface of the tumor. The AJCC 8th edition now uses DOI for staging (29). In our study, DOI failed to retain its significance in the multivariate analysis, possibly due to its association with the T-stage (p-value <0.001 using the Mann-Whitney-Wilcoxon test) causing multi collinearity and due to the small sample size. However, due to the importance of DOI found in other papers, we still conducted a univariate ROC curve analysis of DOI (Table 5), and reported the area under the curve and estimated best cut-off according to our analysis. DOI was found to have a modest predictive value in such a univariate analysis.

The worst pattern of invasion (WPOI) is an important prognostic factor for oral cavity squamous carcinomas (30–32). Five different patterns of WPOI (1–5) have been described (13). Various studies suggested that WPOI 5 is associated with a higher risk of lymph node metastasis compared to the other patterns (WPOI 1-4) in early-stage OSCC (11,33,34). Our findings were also in concordance with these studies. Moreover when measured together with DOI, the chances of predicting the occult metastasis increase. However, some studies have shown discordant results suggesting no effect of WPOI on occult metastasis (26,35).

Lymphovascular invasion (LVI) was also associated with lymph nodal metastasis, with a high odds ratio. However, the predictive effect of LVI was limited by the relatively low number of cases having a positive LVI. The positive cases, however, were associated with a greatly increased risk of metastasis (5 out of 6 cases in our study having LVI had lymph nodal metastasis). Even in the multivariate analysis, even though LVI by itself did not have a significant p-value at an alpha of 0.05.

The T-stage was another important prognostic factor that also retained its importance in multivariate analysis. In do-ing so, it possibly competed against DOI due to significant association and caused the latter to lose its significance. This effect was exacerbated by the small sample size. However, we believe that all the factors that were significant in the univariate analysis are important predictors for nodal metastasis in early-stage OSCC, and information from all the factors should be considered.

It is pertinent to mention that while reporting histopath-ological samples from OSCC these parameters should be evaluated in the standard reporting format as they are associated with a high risk of nodal metastasis. In our study, grade and perineural invasion were not statistically significant to provide any information regarding metastasis.

Multifactorial predictive models and scoring systems have been suggested by authors. These models evaluate multiple histomorphological parameters at different levels to predict metastasis. Multiple variables seem to predict more accurately when evaluated in large-scale studies than individual ones (11,28,36). We found WPOI 5 and T-stage as better predictors in early-stage OSCC, while it was extremely likely that DOI and LVI have a significant association with the same. However, the study was limited due to the small sample size. This led to sparsity of data, which carries a risk of optimistic estimates of odds ratios by ordinary logistic regression. Therefore, we carried out a Firth logistic regression, which is a standard method for reducing small sample bias. The Firth method uses a method known as penalized likelihood (18). A possible alternative would have been to use exact logistic regression, e.g. using the R package “elrm”. However, exact logistic regression is a computationally intensive Monte Carlo Markov chain (MCMC) method. The presence of a continuous variable as a predictor further increases the sparsity, demanding greater computational power. In our case, we did not get valid exact logistic regression results for our multivariate analysis even after 10 million iterations leaving us the choice of using just penalization methods like the Firth logistic regression.

We evaluated some of the histological parameters involved in predicting the nodal metastasis in early-stage OSCC. T-stage, WPOI, DOI, and LVI were the major significant parameters that influenced the nodal metastasis on univariate analysis. Inclusion of these parameters in routine standard reporting will increase the likelihood of predicting the nodal metastasis and hence guiding the clinicians to choose the best treatment protocol for the patients.

## Conflict of Interest

None.
